# Supplementary material for: Two-Dimensional MXene as a Promising Adsorbent for Trihalomethanes Removal: A Density-Functional Theory Study
Source: Nanomaterials (Basel). 2024 Feb 29;14(5):454. doi: 10.3390/nano14050454 (PMC10934524; doi:10.3390/nano14050454)
Supplement: Supplementary file 1 [file nanomaterials-14-00454-s001.zip › Supplementary file .pdf]

Calculation and Computational Details

To assess the reactivity of the models, crucial parameters such as the band gap, transition dipole moment (TDM), and molecular electrostatic potential (MESP) map were computed for each model at the same level of theory. Additionally, the adsorption energy was calculated to investigate the sensitivity and efficiency of MXene functionalization for the removal of tri-halomethanes (THs).The choice of the B3LYP functional and the LANL2DZ basis set for quantum chemical calculations is underpinned by their established efficacy, particularly in simulating MXene structures and their interactions with tri-halomethanes. This selection is motivated by the successful application of these computational methods in prior investigations involving MXene[1–3]. The B3LYP functional, known for its hybrid nature combining Hartree-Fock exchange and density functional exchange-correlation functionals, provides a balanced accuracy for predicting structural and electronic properties across diverse molecular systems [1,4–7]. The LANL2DZ basis set, featuring effective core potentials and tailored for transition metals like titanium, is specifically designed to ensure accurate calculations involving heavy elements, rendering it well-suited for MXene studies [1,4,5]. These methodological choices, validated by previous research, aim to strike a balance between computational efficiency and accuracy, thereby providing a reliable platform for simulating the electronic structure and reactivity of MXene and its interactions with tri-halomethanes. The selection of the LANL2DZ basis set for Ti transition metals is further justified by its effectiveness in describing interactions between organic molecules and transition metal compound clusters, as confirmed in previous studies [2]
